# SIRT3 Regulation of Mitochondrial Quality Control in Neurodegenerative Diseases

**DOI:** 10.3389/fnagi.2019.00313

**Published:** 2019-11-12

**Authors:** Hao Meng, Wan-Yu Yan, Yu-Hong Lei, Zheng Wan, Ye-Ye Hou, Lian-Kun Sun, Jue-Pu Zhou

**Affiliations:** ^1^Department of Pathophysiology, College of Basic Medical Sciences, Jilin University, Changchun, China; ^2^Department of Neurosurgery, The First Hospital of Jilin University, Changchun, China

**Keywords:** mitochondrial NAD-dependent deacetylase sirtuin-3, mitochondrial quality control, signaling pathway, neurodegenerative diseases, neuroprotective effects

## Abstract

Neurodegenerative diseases are disorders that are characterized by a progressive decline of motor and/or cognitive functions caused by the selective degeneration and loss of neurons within the central nervous system. The most common neurodegenerative diseases are Alzheimer’s disease (AD), Parkinson’s disease (PD), and Huntington’s disease (HD). Neurons have high energy demands, and dysregulation of mitochondrial quality and function is an important cause of neuronal degeneration. Mitochondrial quality control plays an important role in maintaining mitochondrial integrity and ensuring normal mitochondrial function; thus, defects in mitochondrial quality control are also significant causes of neurodegenerative diseases. The mitochondrial deacetylase SIRT3 has been found to have a large effect on mitochondrial function. Recent studies have also shown that SIRT3 has a role in mitochondrial quality control, including in the refolding or degradation of misfolded/unfolded proteins, mitochondrial dynamics, mitophagy, and mitochondrial biogenesis, all of which are affected in neurodegenerative diseases.

## Introduction

Neurodegenerative diseases involve mitochondrial dysfunction caused by various factors, which ultimately leads to progressive degeneration, apoptosis, or necrosis of neurons (Srivastava and Yadav, 2016). Degeneration can be selective for specific neuron types, such as in Parkinson’s disease (PD), where selective degeneration of substantia nigra (SN) dopaminergic neurons occurs; in contrast, Alzheimer’s disease (AD) and Huntington’s disease (HD) neuropathology shows extensive neuronal degeneration (Mavroudis et al., 2010; Dexter and Jenner, 2013; Saudou and Humbert, 2016; de Baat et al., 2018).

Mitochondria are important organelles in the nervous system, especially at nodes of Ranvier and axonal ends, and they are usually needed to supply energy to neurons and maintain Ca^2+^-based ion homeostasis through axonal transport (Giorgi et al., 2018). When neurons age, the mitochondrial functions also become abnormal; factors such as increased reactive oxygen species (ROS) can damage mitochondrial oxidative phosphorylation, thereby affecting long-distance axonal transport of mitochondria, leading to synaptic dysfunction and neurodegeneration (Reynolds et al., 2004; Baloh et al., 2007). Therefore, a robust mitochondrial function may be more important for neurons than for other cells (Knott et al., 2008).

Mitochondria have multiple functions in neurons, such as in oxidative phosphorylation, lipid metabolism, amino acid metabolism, and maintenance of Ca^2+^ homeostasis (Pipatpiboon et al., 2012; Johnson et al., 2014; Bergman and Ben-Shachar, 2016; Giorgi et al., 2018). Previous studies have found that a dysregulation of mitochondrial function is an important cause of neuronal degeneration, and recent studies have also shown that mitochondrial quality control has a role in maintaining mitochondrial integrity and ensuring normal mitochondrial function (Palikaras and Tavernarakis, 2014; Ni et al., 2015; Palikaras et al., 2015). Defects in mitochondrial quality control are therefore also important factors in neurodegenerative diseases (Dupuis, 2014; Zorzano and Claret, 2015; Khanna et al., 2019). The currently well-defined mitochondrial quality control pathways include the mitochondrial unfolded protein response (mtUPR), dynamic remodeling and repair of mitochondrial fission and fusion, mitophagy, and mitochondrial biogenesis, which is coordinated with mitophagy (Zhu et al., 2013; Palikaras and Tavernarakis, 2014).

Mitochondrial function is regulated by a variety of enzymes, and in this review, we have focused on the important mitochondrial NAD-dependent deacetylase sirtuin-3 (SIRT3; Finley et al., 2011). SIRT3 belongs to the histone deacetylase family of silent information regulator 2 (Sir2) proteins, or sirtuins, whose deacetylase activity affects the acetylation status of at least 165 proteins in the mitochondrial proteome (Lombard et al., 2007; Schwer et al., 2009; Hebert et al., 2013). SIRT3 has important effects on mitochondrial sugar, fat, and amino acid metabolism; electron transport; oxidative phosphorylation; and oxidative stress (Hiromasa et al., 2004; Lombard et al., 2007; Hallows et al., 2011; Huang et al., 2016; Wang et al., 2019). Recently, studies have also reported that SIRT3 plays an important role in regulating mitochondrial quality control in neuronal mitochondria (Tseng et al., 2013; Samant et al., 2014; Liu et al., 2018).

This review summarizes recent research into mitochondrial quality control and the role of SIRT3 in mitochondrial function, and further illustrates the effects of SIRT3 on mitochondrial quality control in the neurodegenerative diseases AD, PD, and HD. This will provide a reference for exploring the relationship between mitochondrial function and quality, as well as for seeking new targets for the treatment of neurodegenerative diseases.

## Mitochondrial Quality Control

**Figure 1:**
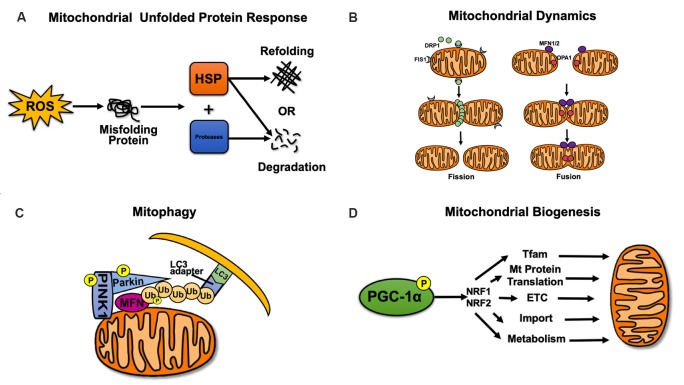
Mitochondrial quality control. **(A)** Mitochondrial unfolded protein response. Refolding or degradation of mitochondrial misfolded/unfolded proteins is mainly mediated by molecular chaperones and proteolytic enzymes in the mitochondria. **(B)** Dynamic remodeling and repair of mitochondrial fission and fusion. Mitochondrial fission is regulated by dynamin-related GTPase (DRP1) and FIS1, while mitochondrial fusion is regulated by MFN1/2 and optic atrophy 1 (OPA1). **(C)** Mitophagy. The PINK1–Parkin-mediated mitophagy pathway can mediate the formation of autophagosomes. **(D)** Mitochondrial biogenesis. Mitochondrial biogenesis is mainly regulated by PGC-1α and nuclear respiratory factors 1 and 2 (NRF1/2).

The normal functions of mitochondria rely on mitochondrial quality, which is regulated by mitochondrial quality control (Palikaras et al., 2015). Refolding or degradation of mitochondrial misfolded/unfolded proteins is mainly mediated by molecular chaperones and proteolytic enzymes in the mitochondria (Bernales et al., 2012; Haroon and Vermulst, 2016). When mitochondria are damaged, excessive ROS production that exceeds the scavenging ability of superoxide dismutase 2 (SOD2) leads to lipid oxidation, mutations or deletions of mtDNA, and protein misfolding (Papa and Germain, 2014). At this point, mitochondria initiate the unfolded protein response (mtUPR) and activate molecular chaperones (such as the Hsp60/Hsp10 complex or the Hsp60–mtHsp70 complex) and proteolytic enzymes (such as Lon proteases; Figure 1A). Molecular chaperones are involved in regulating the refolding or degradation of misfolded proteins (Kao et al., 2015). In addition to degrading misfolded proteins, Lon proteases regulate mtDNA replication and transcription by acting on mitochondrial transcription factor A (TFAM; Matsushima et al., 2010; Kao et al., 2015).

Mitochondrial fission and fusion are collectively referred to as mitochondrial dynamics (Bertholet et al., 2016). In mammals, mitochondrial fission is regulated by dynamin-related GTPase (DRP1) and mitochondrial fission 1 (FIS1). DRP1 is the key protein that controls mitochondrial fission, and FIS1 is considered to be the receptor of DRP1 (Palmer et al., 2013; Singh et al., 2016). The fusion of the mitochondrial outer and inner membranes is mediated by mitofusin1/2 (MFN1/2) and optic atrophy 1 (OPA1), respectively (Figure 1B; Chang and Doering, 2018). Concurrently, OPA1 controls the shape of mitochondria cristae after fusion, which directly affects the stability of electron transport chain (ETC) complexes, especially complex IV, thus indicating that OPA1 regulates mitochondrial fusion while maintaining normal oxidative phosphorylation (Olichon et al., 2006).

Mitophagy selectively removes damaged mitochondria and plays an important role in maintaining normal mitochondrial function (Vigié and Camougrand, 2017). A variety of mitophagy pathways have been identified, including ubiquitin-dependent and ubiquitin-independent pathways. The most widely studied of these pathways is the PINK1–Parkin-mediated mitophagy pathway (Figure 1C; Ding and Yin, 2012). Mitophagy caused by the PINK1–Parkin pathway is widespread in mitochondria (Ziviani and Whitworth, 2010). Phosphatase and tensin homolog (PTEN)-induced putative kinase 1 (PINK1) accumulates in the outer membranes of damaged mitochondria and is activated by autophosphorylation, thus recruiting Parkin to translocate to the outer membrane of the mitochondria. Parkin is phosphorylated by PINK1 to continue the ubiquitination of MFN1/2, voltage-dependent anion channel 1 (VDAC1), and small GTPase Miro, among others. Ubiquitinated MFN1/2 is then degraded by proteasomes, thereby preventing mitochondrial fusion and promoting mitochondrial fission (Poole et al., 2010; Nguyen et al., 2016). Subsequently, the ubiquitin chains on mitochondria can recruit the microtubule-associated protein 1A/1B-light chain 3 (LC3) adaptors P62, OPTN, NDP52, TAX1BP1, and NBR1. LC3 can recognize LC3 adaptors and mediate the formation of autophagosomes to realize mitophagy (Lazarou et al., 2015).

Mitochondrial biogenesis and mitophagy represent two opposing but coordinated processes that determine mitochondrial content, structure, and function (Zhu et al., 2013). Mitochondrial biogenesis includes the replication, transcription, and translation of mtDNA; the synthesis and import of nuclear-encoded mitochondrial protein; the recruitment of newly synthesized proteins and lipids; and the construction of the mitochondrial reticulum (Rasbach and Schnellmann, 2007; Zhu et al., 2013). Peroxisome proliferator-activated receptor γ (PPARγ) coactivator-1α (PGC-1α) is considered the major regulator of mitochondrial biogenesis. It interacts with nuclear respiratory factors 1 and 2 (NRF1, 2), which can activate TFAM (Scarpulla, 2002) and bind to the promoter region of the nuclear gene encoding the five complex subunits of the mitochondrial electron transport chain (ETC), thereby affecting the synthesis of nuclear-encoded mitochondrial proteins and the transcription of mtDNA (Figure 1D; Uittenbogaard and Chiaramello, 2014).

## Regulation of Mitochondrial Function by Sirt3

**Figure 2:**
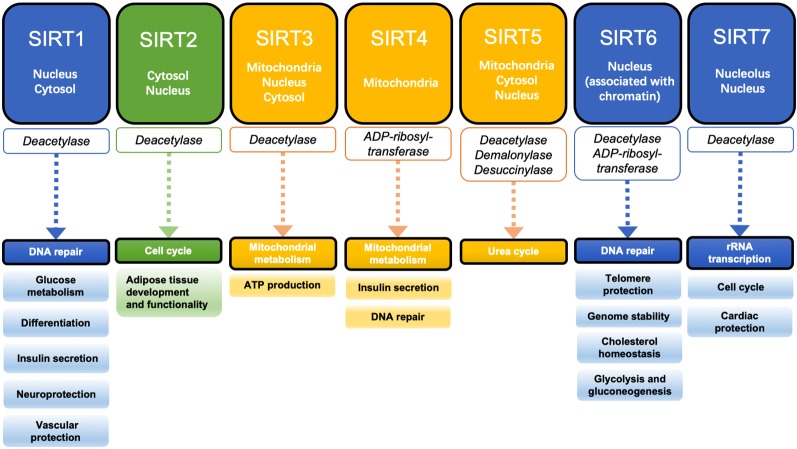
Localization, enzyme activity, and function of the sirtuin family. SIRT1, SIRT6, and SIRT7 are mainly located in the nucleus and regulate DNA repair and gene transcription. SIRT2 is mainly located in the cytosol and plays an important role in the regulation of the cell cycle. SIRT3, SIRT4, and SIRT5 are known as mitochondrial sirtuins and are involved in the regulation of mitochondrial functions.

Sir2 proteins are a family of histone deacetylases that catalyze the deacetylation and ribosylation of histone and non-histone lysine residues (Winnik et al., 2015). They were first discovered to affect energy metabolism through enzyme activity regulation in studies of yeast aging, and they play an important role in promoting health and survival (Haigis and Sinclair, 2010). There are seven mammalian sirtuins (SIRT1–7). SIRT1, SIRT6, and SIRT7 are mainly located in the nucleus and participate in DNA repair and transcription regulation of related genes (Brunet et al., 2004; Grob et al., 2009; Cardus et al., 2013). SIRT2 is mostly located in the cytosol and its main substrate is α-tubulin, which is involved in cell cycle regulation (Li et al., 2007). SIRT3, SIRT4, and SIRT5 are called mitochondrial sirtuins. SIRT3 plays an important role in the metabolism of glycosides, lipids, and amino acids in mitochondria (Hiromasa et al., 2004; Lombard et al., 2007; Scher et al., 2007; Wang et al., 2019). SIRT4 can inhibit glutamate dehydrogenase (GDH) activity through ADP ribosylation, thus reducing fatty acid oxidation (Wang et al., 2018). SIRT5 can regulate the urea cycle by deacetylating to activate carbamyl phosphate synthase 1 (CPS1), and also has demalonylase and desuccinylase activity (Figure 2; Du et al., 2011).

SIRT3 is one of the most important deacetylases in mitochondria, and it plays an important role in regulating mitochondrial function (Finley et al., 2011). For example, deacetylation of the pyruvate dehydrogenase complex (PDC) by SIRT3 during glycolysis allows pyruvate to participate in the Krebs cycle and accelerates glucose uptake by activating protein kinase B (Akt; Hiromasa et al., 2004; Wang et al., 2019). SIRT3 also ensures the normalization of fatty acid β-oxidation by deacetylating long-chain acyl-CoA dehydrogenase (LCAD) and acetyl-CoA synthetase 2 (AceCS2; Schwer et al., 2006; Sakakibara et al., 2009; Hirschey et al., 2010) and plays a role in the formation of ketone bodies by deacetylating 3-hydroxy-3-methylglutaryl-CoA synthetase (HMGCS2; Hirschey et al., 2010; Shimazu et al., 2010). Deacetylation of GDH by SIRT3 promotes amino acid utilization (Lombard et al., 2007). In addition, the key enzyme in the urea cycle, ornithine carbamoyltransferase (OTC), is a substrate of SIRT3 (Hallows et al., 2011). SIRT3 also plays an important role in promoting the normal progression of the tricarboxylic acid (TCA) cycle by deacetylating succinate dehydrogenase (SDH) and isocitrate dehydrogenase (IDH; Cimen et al., 2010; Fritz et al., 2013). Furthermore, the deacetylation by SIRT3 of numerous complex I–V subunits in the oxidative respiratory chain indicates the importance of this enzyme (Ahn et al., 2008 Mattson et al., 2008; Cheng et al., 2016). SIRT3 also prevents or delays damage caused by oxidative stress by activating many antioxidant factors, including FOXO3, IDH2, and SOD (Tseng et al., 2013; Huang et al., 2016; Cui et al., 2017).

## Mitochondrial Quality Control by Sirt3 in Neurodegenerative Diseases

**Figure 3:**
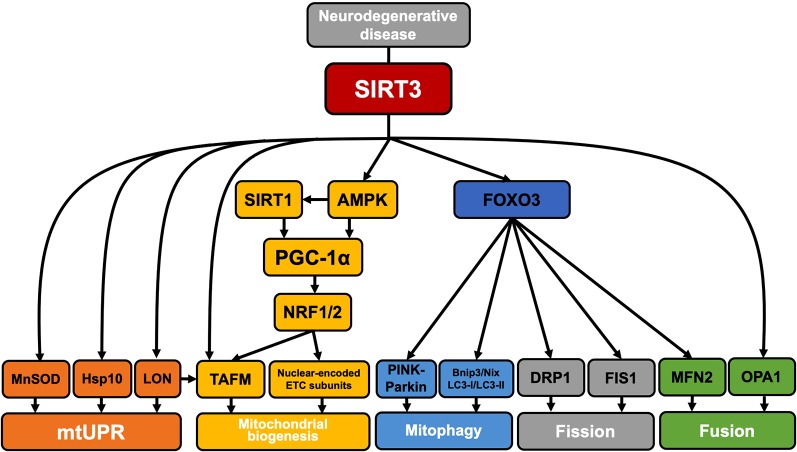
SIRT3 regulation of mitochondrial quality control in neurodegenerative disease. SIRT3 is involved in the regulation of mitochondrial quality control in neurodegenerative diseases. SIRT3 deacetylates MnSOD, HSP10, and Lon proteases and participates in the mitochondrial unfolded protein response. SIRT3 can activate AMPK by upregulating the ratio of AMP/ATP. Activated AMPK can directly phosphorylate PGC-1α or enhance SIRT1 activity by increasing NAD+ levels, and SIRT1 can then deacetylate PGC-1α. PGC-1α interacts with NRF1/2 to activate TFAM and promote the synthesis and import of nuclear-encoded ETC complex subunits [such as the Fe–S subunit of succinate dehydrogenase (SDH) or the subunit of cytochrome c oxidase]. In addition, Lon proteases are also involved in the selective degradation of TFAM to regulate mitochondrial biogenesis. SIRT3 can deacetylate FOXO3, thereby activating PINK1–Parkin pathway-mediated mitophagy. Activated FOXO3 also promotes the expression of Bnip3/Nix, LC3-I/LC3-II, DRP1, FIS1, and MNF2 and regulates mitophagy and mitochondrial fission/fusion. The direct deacetylation of OPA1 by SIRT3 is also involved in the regulation of mitochondrial fusion.

In addition to its role in mitochondrial function, SIRT3 has recently been reported to have an effect on mitochondrial quality control (Tseng et al., 2013; Gibellini et al., 2014). In this part of our review, we studied three major neurodegenerative diseases—AD, PD, and HD—and summarized the regulation of mitochondrial quality control by SIRT3 in each disease (Figure 3).

## Alzheimer’S Disease

AD is the most common form of dementia in the elderly (Barker et al., 2015). In this disease, there is progressive degeneration of vulnerable parts of the central nervous system (mainly the hippocampus and cortex), leading to a decline in cognitive function (Mavroudis et al., 2010). The etiology of AD is associated with impaired brain energy metabolism and oxidative stress, leading to synaptic degeneration and related cognitive deficits (Blass et al., 2002; Dumont and Beal, 2011).

In AD, the accumulation of amyloid β (Aβ) in synapses and synaptic mitochondria is an important cause of synaptic degeneration and cognitive decline in AD patients (Calkins and Reddy, 2011). Accumulation of Aβ disrupts the mitochondrial membrane potential, leading to the production of ROS, and activates the mitochondrial fission-associated proteins DRP1 and FIS1, causing mitochondria to divide excessively. Defective mitochondria cannot move to the synapse to provide ATP, which ultimately causes synaptic degeneration, leading to neuronal degeneration (Calkins and Reddy, 2011; Kapogiannis and Mattson, 2011). Upregulation of SIRT3 expression in AD not only reduces ROS damage to mitochondrial structure by deacetylation and activation of SOD2 (Jacobs et al., 2008; Cho et al., 2009), but also participates in the regulation of mitochondrial quality (Kincaid and Bossy-Wetzel, 2013). SIRT3 can deacetylate the inner membrane fusion protein OPA1 and increase its GTPase activity, and can also promote gene expression of the outer membrane fusion protein MFN2 through activation of FOXO3, thereby slowing the excessive mitochondrial division caused by abnormal DRP1 and FIS1 activity (Kincaid and Bossy-Wetzel, 2013; Ribeiro et al., 2019). This suggests that SIRT3 acts to regulate the balance of mitochondrial division and fusion in neurons, thereby preventing or slowing the damage and degeneration of neuronal axons that occurs because mitochondrial fragmentation leads to insufficient ATP supply (Knott et al., 2008; Tseng et al., 2013; Samant et al., 2014).

In addition, studies have shown that expression levels of PGC-1α, NRF1, NRF2, and TAFM are all significantly decreased in the hippocampus of AD patients, indicating that impaired mitochondrial biogenesis likely contributes to mitochondrial dysfunction in AD (Sheng et al., 2012). SIRT3 has been reported to promote mitochondrial biogenesis by promoting PGC-1α expression (Fu et al., 2012). SIRT3 activates AMP-activated protein kinase (AMPK) by activating AceCS2 to increase the AMP/ATP ratio (Hallows et al., 2006). Activated AMPK can directly phosphorylate PGC-1α (Jäger et al., 2007) or enhance SIRT1 activity by increasing NAD+ levels. SIRT1 can then deacetylate PGC-1α, thereby promoting mitochondrial biogenesis and delaying AD progression (Cantó et al., 2009).

## Parkinson’S Disease

PD is one of the most common neurodegenerative diseases and is characterized by the preferential, progressive degeneration of dopaminergic neurons in the SN pars compacta and a loss of striatal dopamine input (Dexter and Jenner, 2013). The pathogenic mechanisms of PD are presumed to include mitochondrial dysfunction and abnormal changes in mitochondrial quality (Mandemakers et al., 2007).

PINK1 is a mitochondrial Ser/Thr kinase, and a loss or mutation of PINK1 has an important impact on PD pathogenesis. Mutations in PINK1 can promote mitochondrial fission or reduce mitochondrial fusion in mammalian cells. Mutant PINK1 promotes mitochondrial translocation of DRP1 and reduces the degradation of DRP1 and FIS1 by Parkin, resulting in increased fission and damage to mitochondria (Deng et al., 2008). Studies have shown that SIRT3 can act indirectly on PINK1, and the SIRT3–FOXO3 pathway activates mitophagy *via* the PINK1–Parkin pathway (Das et al., 2014). In addition, SIRT3 deacetylation of FOXO3 promotes the expression of a variety of FOXO3-dependent genes that are required for mitochondrial homeostasis. Studies have shown that the SIRT3–FOXO3 pathway induces the expression of DRP1, FIS1, and MFN2 to coordinate mitochondrial fission/fusion, and increases Bnip3, Nix, and LC3-II/LC3-I for mitophagy in the presence of mitochondrial damage, resulting in the degradation of damaged mitochondria (Tseng et al., 2013). This type of regulation delays the degeneration and necrosis of dopaminergic neurons resulting from the mitochondrial damage caused by mutant PINK1. SIRT3 can, therefore, regulate mitochondrial quality control by increasing the levels of mitochondrial autophagy, thus playing a neuroprotective role (Huang et al., 2019). In addition, PINK1 also has an effect on mitochondrial function. Studies have shown that PINK1 may maintain the stability of the ETC by maintaining Ser250 phosphorylation in the ETC complex I subunit NdufA10 (Gautier et al., 2008; Wood-Kaczmar et al., 2008; Morais et al., 2014), whether SIRT3 can affect mitochondrial function through PINK1 still needs related research reports.”

The accumulation of α-synuclein is also an important neuropathological characteristic of PD. Studies have shown that SIRT3 has neuroprotective effects in a mutant α-synuclein rat model of PD, which may be achieved by enhancing mitochondrial bioenergetics and reducing mitochondrial oxidative stress (Gleave et al., 2017). Studies have also shown that SIRT3 downregulation in the presence of α-synuclein accumulation is accompanied by increased phosphorylation of AMPK, cAMP-response element-binding protein (CREB), and DRP1, as well as decreased levels of OPA1. These results imply impaired mitochondrial dynamics, further supporting the protective role of SIRT3 in relevant PD pathways (Park et al., 2018).

## Huntington’S Disease

HD is a late-onset autosomal dominant neurodegenerative disease caused by repeated amplification of the CAG trinucleotide in the gene encoding the ubiquitin-expressing protein Huntingtin (HTT). In addition to central nervous system dysfunction, which includes neuronal cell death in the striatum and cortex, cell dysfunction in peripheral tissues is also widespread in HD (Saudou and Humbert, 2016).

In HD patients, mitochondrial ETC complex II, III activity is decreased, and aconitase activity in the basal ganglia is also reduced. This also occurs in striatal cells of mutant Huntingtin knock-in mice: mitochondrial oxidative phosphorylation and ATP production are both severely damaged, resulting in the massive production of ROS (Browne and Beal, 2004). Accumulation of ROS leads to mtDNA damage and the misfolding of proteins (Papa and Germain, 2014). SIRT3 plays an important role in repairing misfolded mitochondrial proteins, and SIRT3 is a major coordinator of mitochondrial unfolded protein response (mtUPR); for example, SIRT3 can reduce ROS levels by deacetylating MnSOD (Shi et al., 2017). Molecular chaperones (such as the Hsp60/Hsp10 complex and the Hsp60–mtHsp70 complex) participate in mtUPR to achieve the refolding or degradation of misfolded/unfolded proteins (Bie et al., 2016; Yadav et al., 2019). Studies have shown that the decline of HSP60 leads to mitochondrial dysfunction in HD, HSP70 is involved in the regulation of misfolded proteins in HD, suggesting that molecular chaperones play a neuroprotective role in HD (Wacker et al., 2009; Reis et al., 2017; Fu et al., 2019). Hsp10 has been confirmed as a deacetylated substrate for SIRT3 (Lu et al., 2014). In addition, immunolocalization revealed that Lon protease is a substrate of SIRT3, and SIRT3 activates Lon protease by deacetylation (Gibellini et al., 2014). Activated Lon protease degrades oxidized aconitase in HD and regulates mtDNA replication and transcription by acting on TFAM, thereby preventing mitochondrial dysfunction resulting from the massive accumulation of damaged proteins, which can cause neuronal damage (Goo et al., 2014; Neo and Tang, 2018). TFAM is also a direct deacetylation substrate for SIRT3, and SIRT3 promotes mitochondrial biogenesis by deacetylating to enhance TFAM expression (Liu et al., 2018).

Furthermore, studies have shown that AMPK activation can resist mutated HTT-induced cytotoxicity in the early stage of HD (Vázquez-Manrique et al., 2016). However, as the aging of organisms or the less ability of cells to cope with proteotoxic stress and its consequences, AMPK may become abnormally active due to the overwhelming quantity of stress signals, and its activity may be fatal to cells (Ju et al., 2014). SIRT3 has been found to activate AMPK in HD by deacetylating liver kinase B1 (LKB1) to regulate mitochondrial biogenesis and energy metabolism homeostasis, further demonstrating that SIRT3 exerts neuroprotective effects in HD (Fu et al., 2012).

In addition, mutant HTT stimulates the activation of DRP1 and FIS1 because it has a higher affinity than that of wild-type HTT. Mutant HTT also leads to decreased MFN1/2 and OPA1 expression, thus causing high levels of mitochondrial fission and low levels of mitochondrial fusion, which leads to disordered mitochondrial dynamics (Kim et al., 2010; Song et al., 2011; Jodeiri Farshbaf and Ghaedi, 2017). Direct deacetylation of OPA1 by SIRT3 can increase mitochondrial fusion levels, and deacetylation of FOXO3 by SIRT3 can promote *MFN2* expression, thereby delaying the disorder of mitochondrial fission and fusion, maintaining mitochondrial function and axonal transport, and slowing the progression of striatal lesions (Tseng et al., 2013; Samant et al., 2014).

## Future Directions

Several new pathways of mitochondrial quality control have been reported recently; for example, mitochondria form mitochondrial-derived vesicles (MDV) under oxidative stress. These vesicles contain oxidized proteins that germinate from damaged mitochondria and dissolve in lysosomes, thus selectively degrading damaged mitochondrial contents and maintaining mitochondrial function (Soubannier et al., 2012; McLelland et al., 2014). Another pathway involves the formation of mitochondrial spheroids, independent of the classical mitophagy pathway (Ding et al., 2012). This suggests that there may be a variety of indefinite mechanisms involved in the regulation of mitochondrial homeostasis as part of mitochondrial quality control. In addition, both MDV and mitochondrial spheroids show a correlation with PINK1 and Parkin (Ni et al., 2015). Whether SIRT3 is involved in the regulation of mitochondrial homeostasis still requires further study.

Although there have been many studies into the role of SIRT3 in the nervous system, many potential mechanisms are still unclear. For example, when studying the neurotrophin pituitary adenylate cyclase-activating polypeptide (PACAP), it was found that PACAP can induce SIRT3 expression and protect neurons against Aβ toxicity. Experiments have also shown that PACAP expression is decreased in an AD animal model and SIRT3 expression is also decreased (Han et al., 2014), which contradicts a previously reported increase in SIRT3 mRNA expression in AD (Weir et al., 2012). These results suggest that the role of SIRT3 needs to be further explored in future research. In addition, it has been reported that SIRT3 regulates mitochondrial ceramide biosynthesis through the deacetylation of ceramide synthase (CerS) 1, 2 and 6 (Novgorodov et al., 2015), and the content of mitochondrial ceramide is closely related to mitochondrial dysfunction and ischemic stroke (Novgorodov and Gudz, 2011), suggesting that the role of SIRT3 in the nervous system remains controversial and needs further study.

Recently, studies have explored the function of SIRT3 by using SIRT3 inhibitors, the most widely used of which is 3-(1H-1,2,3-triazole-4-yl)pyridine (3-TYP), a selective SIRT3 inhibitor. At present, 3-TYP is mainly used to study the regulation of SIRT3 on mROS homeostasis and autophagic flux, which is reflected in the study of ischemia-reperfusion injury and some drugs (such as genipin and melatonin; Pi et al., 2015; Zhai et al., 2017; Shumin et al., 2018; Ouyang et al., 2019). 3-TYP has also been used to study the relationship between some upstream factors (including PGC-1α and SIRT1) and SIRT3 (Feng et al., 2019; Liu et al., 2019), as well as the regulation of mitophagy by SIRT3 (Wang et al., 2019). As a new method, SIRT3 inhibitors may play an important role in future research into SIRT3.

In addition to the role of SIRT3 in mitochondrial quality control in neurodegenerative diseases, recent studies have gradually started to investigate the roles of two other mitochondrial sirtuins, SIRT4 and SIRT5, in mitochondrial quality control. SIRT4 promotes mitochondrial fusion by interacting with OPA1 in an enzymatically dependent manner (Lang et al., 2017) and reduces the recruitment of DRP1 to the mitochondrial membrane by inhibiting DRP1 phosphorylation, thereby reducing mitochondrial fission and mitophagy (Fu et al., 2017). SIRT5 has also been found to regulate mitochondrial protein degradation by mitophagy in challenging metabolic conditions (Guedouari et al., 2017). These results suggest that future studies of mitochondrial quality control may need to focus more on the interrelationships and interactions among these three mitochondrial sirtuins.

SIRT1 is also involved in regulating mitochondrial quality control. The AMPK–PGC-1α axis and SIRT1–PGC-1α axis are the two main pathways that regulate mitochondrial biogenesis. AMPK can activate PGC-1α by phosphorylation (Jäger et al., 2007) and enhance the activity of SIRT1 by increasing cellular NAD+ levels. SIRT1 can then activate PGC-1α by deacetylation, thus initiating mitochondrial biogenesis (Cantó et al., 2009).

## Conclusions

Based on the roles of SIRT3 in mitochondrial function, we further reviewed the involvement of SIRT3 in mitochondrial quality control in the nervous system, and especially in neurodegenerative diseases. We reviewed that SIRT3 has an effect on mitochondrial quality control through the mitochondrial unfolded protein response, mitochondrial dynamics, mitophagy, and mitochondrial biogenesis. The role of SIRT3 in mitochondrial function and quality control suggests that changes in mitochondrial quality have an important impact on mitochondrial function and that the two work together to maintain mitochondrial homeostasis. Although there have been many studies on the role of SIRT3 in the nervous system, many potential mechanisms are still unclear and need to be further explored in future research.

## Author Contributions

W-YY and Y-HL searched and classified the references. ZW and Y-YH created the figures. L-KS and J-PZ designed and supervised this review. HM wrote this article.

## Conflict of Interest

The authors declare that the research was conducted in the absence of any commercial or financial relationships that could be construed as a potential conflict of interest.
